# Identification of disease-specific motifs in the antibody specificity repertoire via next-generation sequencing

**DOI:** 10.1038/srep30312

**Published:** 2016-08-02

**Authors:** Robert J. Pantazes, Jack Reifert, Joel Bozekowski, Kelly N. Ibsen, Joseph A. Murray, Patrick S. Daugherty

**Affiliations:** 1 Department of Chemical Engineering, University of California, Santa Barbara, CA 93106, USA; 2 Serimmune, Inc, Santa Barbara, CA 93105, USA; 3 Division of Gastroenterology and Hepatology, Mayo Clinic, Rochester, Minnesota 55905, USA

## Abstract

Disease-specific antibodies can serve as highly effective biomarkers but have been identified for only a relatively small number of autoimmune diseases. A method was developed to identify disease-specific binding motifs through integration of bacterial display peptide library screening, next-generation sequencing (NGS) and computational analysis. Antibody specificity repertoires were determined by identifying bound peptide library members for each specimen using cell sorting and performing NGS. A computational algorithm, termed Identifying Motifs Using Next- generation sequencing Experiments (IMUNE), was developed and applied to discover disease- and healthy control-specific motifs. IMUNE performs comprehensive pattern searches, identifies patterns statistically enriched in the disease or control groups and clusters the patterns to generate motifs. Using celiac disease sera as a discovery set, IMUNE identified a consensus motif (QPEQPF[PS]E) with high diagnostic sensitivity and specificity in a validation sera set, in addition to novel motifs. Peptide display and sequencing (Display-Seq) coupled with IMUNE analysis may thus be useful to characterize antibody repertoires and identify disease-specific antibody epitopes and biomarkers.

The antibody repertoire is a rich source of biomarkers of infectious and autoimmune diseases. Antibodies binding to specific protein and peptide antigens have yielded clinically validated diagnostic tests for autoimmune diseases including celiac disease^1^ (CD), Grave’s disease^2^, rheumatoid arthritis^3^, and type I diabetes^4^. Despite much progress, many existing assays possess low sensitivity and specificity^5^,^6^,^7^,^8^ and many autoimmune disorders lack effective biomarkers.

Many antibody biomarkers have been identified through extensive fundamental research into the particular human tissues targeted by the immune response (e.g., small intestine, thyroid, joint synovium and pancreas). Because such approaches are inherently difficult, time consuming and biased, most recent efforts have circumvented these issues by using either protein array^9^,^10^,^11^,^12^,^13^,^14^,^15^ or peptide display^16^,^17^,^18^,^19^,^20^,^21^ technologies. Each approach has limitations. Protein arrays, even whole proteome arrays, remain inherently biased to only identifying hits in the pre-selected structures and cannot encompass the much larger number of environmental and commensal antigens that can induce B-cell responses. Large random peptide display libraries have rarely identified human or environmental antigens associated with disease. The inability to adequately characterize the breadth of peptides selected to bind to an antibody repertoire has limited the extent and quality of information derived from peptide display methods^22^.

There exists a need for methods to identify disease-specific antibodies that may bind to diverse antigens from the microbiome and environment in an unbiased manner without significant loss of useful information. Toward this end, we sought to develop a method to comprehensively analyze human antibody specificity repertoires for biomarker discovery using bacterial peptide display libraries and next-generation sequencing (NGS), termed Display-Seq. Although motif discovery in DNA and protein sequences has been extensively investigated^23^,^24^,^25^,^26^,^27^,^28^,^29^,^30^, existing methods were not suitable to address this problem. Therefore we developed a novel computational algorithm for Identifying Motifs Using Next-generation sequencing Experiments (IMUNE). Celiac Disease (CD) is an autoimmune disorder characterized by antibodies to deamidated gliadin and transglutaminase-2, both of which are highly sensitive and specific biomarkers^31^. The well-characterized nature of this disease motivated its selection to validate the efficacy of coupling Display-Seq with the IMUNE algorithm. Our results demonstrate that IMUNE analysis of Display-Seq datasets enables the rapid discovery of disease-specific antibody epitopes and provides new opportunities for biomarker discovery and molecular diagnostics development.

## Results

### Method overview

**Figure 1:**
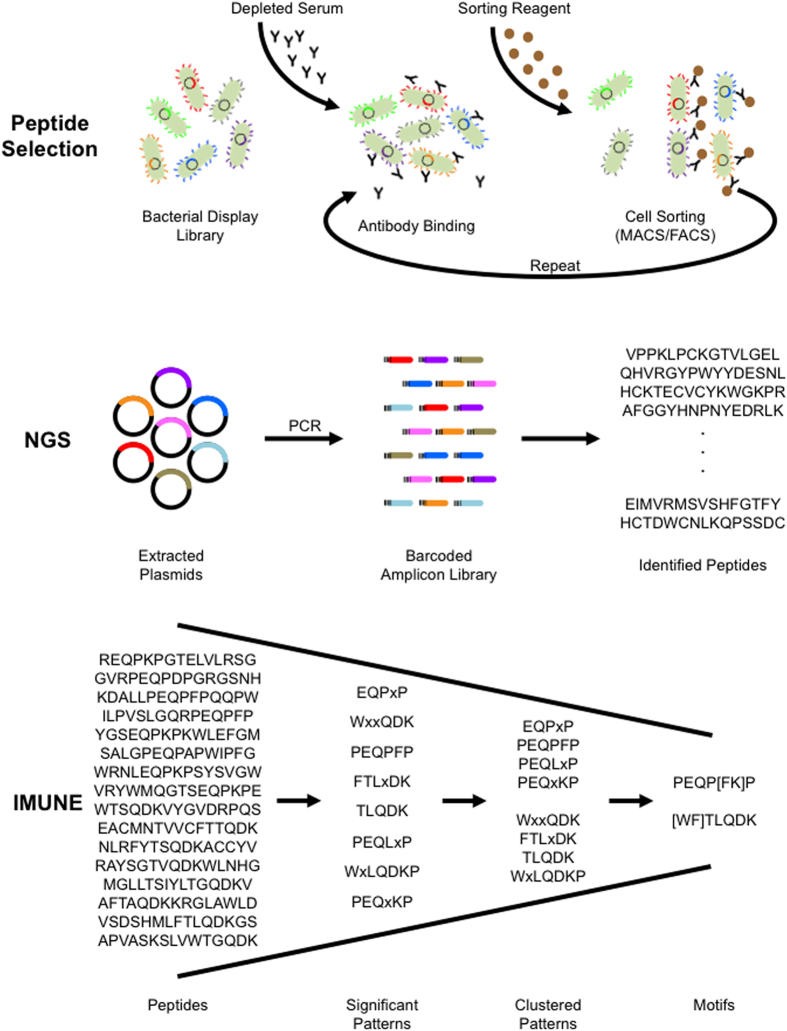
Disease specific antibody epitope discovery using Display-Seq and the IMUNE algorithm. Display-Seq and IMUNE combine experimental screening and next-generation sequencing (NGS) with computational analysis to identify putative disease biomarker motifs. Bacterial display peptide library members that bind antibodies in individual samples are separated using magnetic and/or fluorescence based sorting. NGS is used to identify the peptide sequences composing the binder populations. Patterns of amino acids that are highly statistically significant (e.g. *p* < 0.0001) within the peptides from many disease patients but are not statistically significant (e.g. *p* > 0.05) in control samples are then identified using computational analysis (IMUNE). Finally, similar patterns are clustered together into the putative disease specific biomarker motifs.

To identify antibody binding specificities (i.e. motifs) associated with disease, we integrated an experimental approach using bacterial display peptide libraries and NGS (i.e. Display-Seq) with computational motif discovery (i.e. IMUNE) (Fig. 1). Briefly, in Display-Seq, cell sorting is used to enrich a bacterial display peptide library for binders to antibodies in each individual serum specimen. The set of unique peptides binding to each serum antibody repertoire is then determined using NGS of bar-coded amplicon libraries prepared from the separately enriched peptide libraries. To extract the disease-specific information from these immense datasets, the IMUNE algorithm searches for amino acid *patterns*, where a pattern is a series of defined amino acids possibly interspersed with undefined residues. Tens of millions of peptides binding to antibodies from individuals with disease and unaffected controls are analyzed to identify patterns that are statistically significantly different between the two populations. IMUNE then clusters similar patterns together to generate predicted biomarker motifs.

Display-Seq was applied to sera from 16 CD and 13 healthy control (HC) individuals using a library of 8 × 10^9^ random 12-mer peptides displayed on *Escherichia coli* (*E. coli*) sorted via two rounds of magnetic-activated cell sorting (MACS) as described^21^,^32^. After sorting, greater than 89% of the cell population contained antibody binders, as measured by flow cytometry (Supplemental Fig. 4). Amplicon libraries were prepared for each patient for NGS using the NextSeq instrument (Illumina). A total of 5–14 × 10^6^ reads, representing 2–5 × 10^6^ unique peptides were obtained per patient (Supplemental Table 1).

The IMUNE algorithm was utilized to analyze the Display-Seq data to generate motifs from patterns that are i) significant in at least 50% of the CD patients and not significant in 100% of the HC samples, ii) significant in 50% of the HC samples and not significant in 100% of the CD patients and iii) significant in at least 80% combined of the HC and CD samples. The patterns contained three to five defined positions and had a maximum length of ten total residues, corresponding to 4.2 × 10^8^ total patterns. The definitions of significance in each case are described in the methods and the results are summarized in Supplemental Table 2. IMUNE analysis was completed in 9 hours on a 3.60 GHz Intel Core i7-4790 CPU.

To determine significance, IMUNE compares actual observations of a pattern or motif to expected observations, where expected observations are calculated assuming that positions are independent of one another and using the amino acid frequencies observed in all peptide sequences obtained for each sample. The comparison can take two forms: enrichment (i.e., the ratio of actual observations to expected observations) or the probability according to the Poisson distribution that a number of observations occurred by random chance with the corresponding expected value. Given the library construction (Online Methods) and a lower limit of 1.5 × 10^6^ unique sequences (Supplemental Table 1), motifs and patterns were limited to using no more than five defined positions to ensure that patterns and motifs had expected observations of at least one (Supplemental Note 1). Therefore, although IMUNE identified motifs with more than five positions, the reported enrichment values are based on using the five most enriched, possibly non-contiguous positions.

### Identification of CD-specific motifs

**Figure 2:**
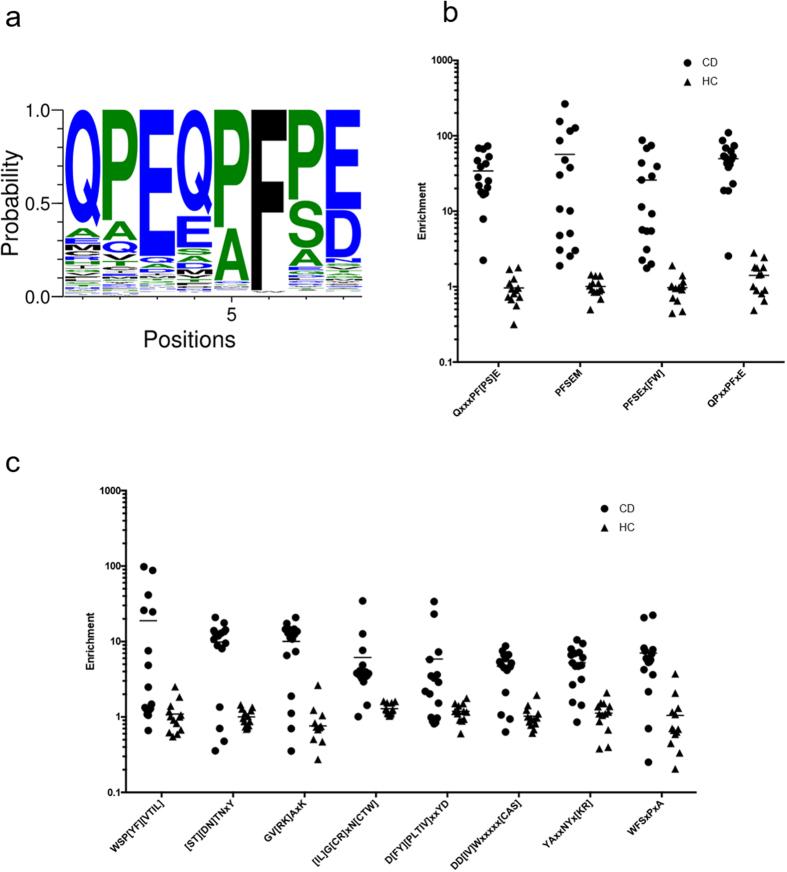
Celiac disease specific motifs identified using IMUNE. The vast majority of motifs that IMUNE identified as being CD sensitive and specific are versions of this eight amino acid motif (**a**). Numerous variations of the motif are highly enriched in many CD samples and have low enrichments in all HC samples (**b**) and every CD sample is significantly enriched in multiple versions of the motif. IMUNE also identified eight other motifs (**c**) that show notable differences in enrichments between many of the CD and HC samples.

IMUNE analysis of Display-Seq datasets identified a prominant eight residue motif: QPEQPF[PS]E (Fig. 2a). The amidated versions (i.e., E → Q) of this motif, QPQQPFPQ and QPQQPFSQ, appear in grain antigens associated with CD: α-, γ-, and ω-gliadins from wheat, γ-, B and C-hordeins from barley, and γ-prolamin, γ-gliadin, and 75 k γ-secalin from rye^33^. Numerous similar versions of this motif are highly enriched (i.e. >10) in the sera many of the 16 CD patients and have low enrichments (i.e. <3) in all of the HC samples. As expected, different CD sera show varying levels of enrichment for different combinations of five residues or interchanging the P and S at the seventh position, but overall every CD patient is highly enriched for this motif. Figure 2b shows four motif variations. Every CD repertoire has an enrichment of at least 15 (minimum value is 18.6) in at least one of the motifs and at least moderate enrichment (i.e. between 5 and 10, minimum value of 7.9) in at least one other motif while no HC sample has an enrichment above 5 (maximum value of 2.8) in any motif.

IMUNE also identified eight additional motifs moderately to highly enriched in many CD patients and not enriched in any HC samples (Fig. 2c). Unlike the prominent motif, these motifs did not contain sequence information that was easily connected to known Celiac antigens. Nonetheless, they still have notably different enrichment profiles in subsets of the CD patients compared to the HC samples.

### Motifs exhibiting enrichment in healthy controls

**Figure 3:**
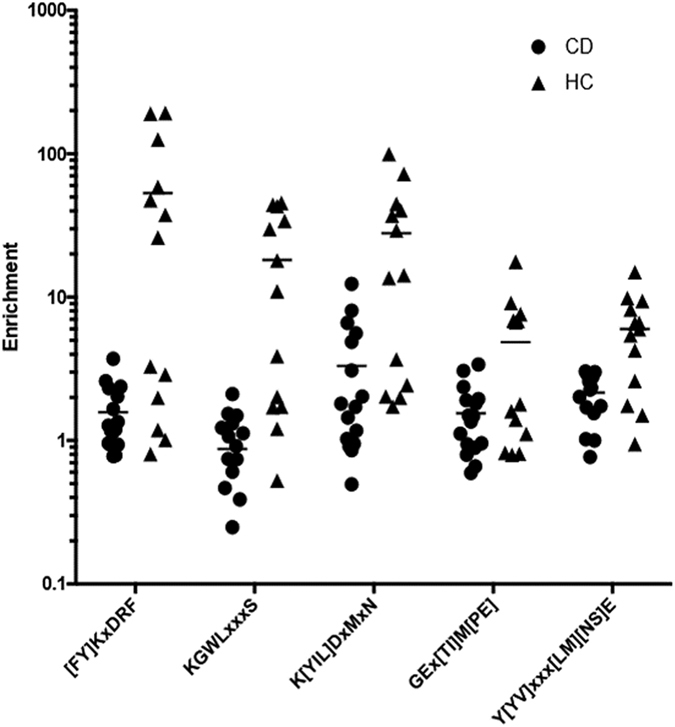
Control specific motifs identified by IMUNE. IMUNE identified five motifs that show higher enrichments in many HC samples compared to the CD patients. These motifs are similar to the 8 non-gliadin CD motifs in that only a subset, rather than nearly all, of the HC samples are much more enriched than the CD patients.

To investigate whether the *absence* of certain antibody specificities might be associated with CD, IMUNE was used to identify motifs enriched by HC but not CD sera. Compared to the CD results, approximately an order of magnitude fewer patterns and motifs were identified as being HC sensitive and specific (Supplemental Table 2). This is consistent with the expectation that the absence of particular antibodies is not associated with CD. The five identified motifs are qualitatively similar to the eight non-gliadin CD motifs, in that only a subset of the HC samples, rather than nearly all of them, have much higher enrichments than the CD patients (Fig. 3). These motifs were not readily associated with environmental antigens using database searches.

### Validation of the CD and HC group specific motifs

**Figure 4:**
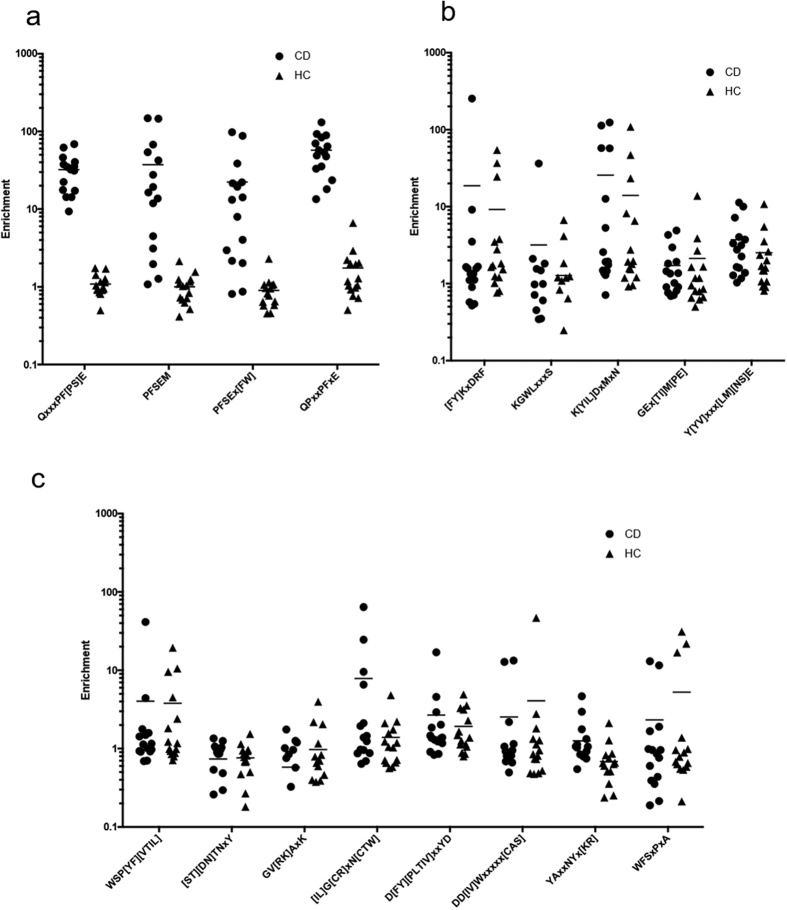
Validation of the CD and HC motifs. Of the motifs identified by IMUNE as being CD or HC specific and sensitive in the discovery samples, only the variants of QPEQPF[PS]E remained so in 15 CD and 15 HC validation samples (**a**). In contrast, the HC (**b**) and remaining CD (**c**) motifs did not show differences in the enrichments between the two populations.

To determine whether CD or HC motifs could serve as effective biomarkers using Display-Seq data, Display-Seq was applied to an additional 15 CD and 15 HC sera. A total of 7–12 × 10^6^ reads representing 2–4 × 10^6^ unique peptides for each sample were obtained (Supplemental Table 3). The unique peptide sequence datasets were then evaluated for the enrichments of nine CD and five HC motifs (Fig. 4). The motifs that are versions of the dominant motif in the CD discovery set (i.e. QPEQPF[PS]E) show enrichments in the validation samples equivalent to those observed in the discovery samples. That is, every CD sample has an enrichment above 16 in at least one motif and is moderately enriched (>9.3) in at least one additional motif. In contrast, one HC sample had an enrichment of 6.6 in one of the four motifs and the next highest enrichment of any HC sample in any motif was 2.9. Thus, every CD sample is more highly enriched in at least two of the four motifs than any HC sample is for any of the motifs. The remaining eight CD and all five HC motifs were not differentially enriched between the CD and HC samples in the validation set. Thus, only the deamidated gliadin-like motifs were validated as highly sensitive and specific biomarkers and the other motifs, including the absence of HC motifs, did not validate as biomarkers.

### Common Celiac Disease and Healthy Control Motifs

**Figure 5:**
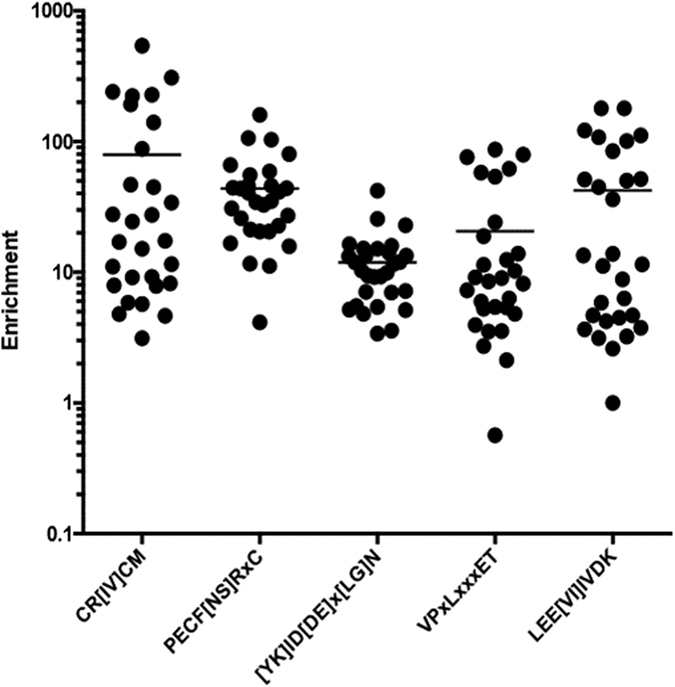
Selected motifs common to 29/29 discovery samples. IMUNE identified nearly 70,000 patterns that were significantly enriched in at least 80% of the CD *and* HC samples. Not surprisingly, many motifs constructed from these patterns were moderately to highly enriched in all 29 of the discovery samples.

To identify motifs common to CD and HC samples, IMUNE was used to construct motifs from patterns significantly enriched in at least 80% of the CD and HC discovery samples. Nearly six-fold more patterns were identified as meeting these criteria than were identified as being specific and sensitive to the CD or HC samples combined, resulting in the identification of at least 315 non-redundant motifs. Unlike the CD results, which were predominantly variants of a single motif, the motifs assembled from these common patterns are highly variable. Many of them are moderately to highly enriched in all 29 discovery samples (Fig. 5). An exhaustive analysis of these motifs is outside the scope of this work, but one motif, VPxLxxxET, matched a B-cell epitope of enterovirus B^34^ and another, LEE[VI]IVDK occurs within the viral coat protein 1 (VP1) of human rhinoviruses. Thus, the IMUNE algorithm successfully identified numerous motifs that are sensitive to every sera sample used for discovery in this study, including those in common environmental antigens.

### Motif discovery in Display-Seq datasets using MEME

Alternative methods of analyzing the identified Display-Seq peptides exist. One approach would be to use whole peptides that were bound by all CD patients and no HC samples, but no such peptides were identified in this study. Another approach is to use the common motif discovery program MEME^27^ to find motifs in peptides that are bound by many CD samples and no HC samples. MEME analysis of the 4984 peptides in the Display-Seq data of the most CD samples and no HC samples required 1 day and 16 hours to complete on the same computer used for the IMUNE analysis. Given that MEME run times scale worse than quadratically with the number of sequences, it is not feasible to run MEME on the full set of tens of millions of peptides identified by Display-Seq.

The MEME analysis identified 16 motifs with E-values less than 0.05 (Supplemental Figs 1–3). Of these motifs, four are variants of the dominant IMUNE motif of QPEQPF[PS]E. These versions of the motif are less sensitive than those identified by IMUNE, with multiple CD samples lacking at least one highly and one moderately enriched version (Supplemental Fig. 1). Of the remaining 12 motifs, six are similar to the eight non-gliadin CD motifs identified by IMUNE, with motifs WSP[YF][VTIL] and D[FY][PLTIV]xxYD not found by MEME. The MEME motifs generally have lower CD, and higher HC enrichments, but are substantially similar in sequence content (Supplemental Fig. 2). The final six motifs discovered by MEME are either not significantly enriched in many (>25%) CD samples or are highly enriched in two or more HC samples (Supplemental Fig. 3). Thus, during a four-fold longer run time, MEME analyzed 3 - 4 *orders of magnitude* fewer peptides than IMUNE, identified several false positive motifs in the discovery set and failed to find gliadin-like motifs that were as specific and sensitive as those discovered by IMUNE.

## Discussion

While the antibody repertoire is a proven source of effective biomarkers, particularly for infection and autoimmunity, the development of robust methods to discover novel disease-specific antibodies remains an area of active investigation^35^. Even though the concept of discovering antibody biomarkers through repertoire profiling or fingerprinting using peptide display libraries was introduced more than 20 years ago^36^,^37^, few clinically validated biomarkers have emerged. The complexity of the antibody repertoire, polyclonal specificities and disease heterogeneity have consistently interfered with biomarker discovery efforts using display libraries and microarrays^38^. In an effort to overcome these challenges, we have developed the Display-Seq method and IMUNE algorithm. Together, they harness NGS to determine the composition of enriched peptide display libraries and identify antibody binding motifs within these large sequence datasets. Using sera from individuals with celiac disease (CD) as a test case, this approach substantially simplified and accelerated the discovery of antibody binding epitopes when compared to reported approaches.

Remarkably, Display-Seq coupled with the IMUNE algorithm directly identified a complete, validated immunogenic epitope. Specifically, QPEQPF[PS]E appears to be a deamidated version of QPQQPF[PS]Q, present in numerous grain antigens associated with Celiac disease. Prior studies using phage or bacterial display with individual or pooled sera identified motifs with four or five consensus positions. These prior approaches required experimental evaluation of many peptide variants^39^ or application of cycles of directed evolution^21^ to identify sensitive and specific extended epitopes. In contrast, the method presented here identified preferred amino acids at eight positions, thereby directly linking the epitope to the corresponding environmental antigens. The computational calculation of enrichment was limited to using no more than five (possibly non-sequential) amino acids in a motif, making it difficult to directly address reactivity of each sample to the extended gliadin-like consensus motif identified in this study. Nonetheless, four motif variants were identified such that every CD sample had enrichments >15 in one motif and >5 in a second and only one HC sample had an enrichment >5 in any motif. In summary, using the enrichments of four motifs, it was possible to accurately classify all 31 CD and 28 HC sera.

The IMUNE algorithm was developed to assemble motifs using information that was computationally identified as disease specific. Although motif identification in biological sequences has been studied for decades^27^, most methods are focused on identifying transcription factor binding sites in DNA^23^. We were unable to identify a motif discovery algorithm that could determine motifs present in one large population of sequences *and* absent from another population, motivating the development of the IMUNE algorithm. Previous peptide display methods removed peptides bound by control patient antibodies with experimental selection steps^16^,^17^,^18^,^21^,^39^, risking the loss of information due to bias introduced by phage propagation^40^,^41^. Additionally, such methods are unlikely to identify antibodies common to all patients but with higher titers in disease. By identifying disease-specific patterns computationally, the IMUNE algorithm both simplifies the experimental procedure and does not risk the information losses inherent to additional selections. The advantage of using an expanded dataset is evident in the comparison of the motifs identified by IMUNE and MEME. MEME was used to analyze ~5000 sequences bound by antibodies in the most CD samples and none of the HC samples, which are those that are most likely to have been selected using previous methods. During a four-fold longer run time, MEME did identify many motifs similar to those found by IMUNE, but it also identified several false positive motifs that were not sensitive and specific to the discovery set, missed at least one candidate biomarker motif found by IMUNE and failed to identify gliadin-like motifs that were sufficiently sensitive and specific to correctly assign the disease state to all 59 samples analyzed in this study.

Both Display-Seq and the IMUNE algorithm have been made possible by recent technological advances. Tens of millions of unique peptides were used here, and the expansive capacity of NGS instrumentation (e.g., Illumina NextSeq500 and HiSeq) may allow hundreds of millions of peptides to be used in future studies. The increasing capabilities of NGS now make it feasible to perform antibody biomarker discovery using millions of peptides for tens or hundreds of patients. Similarly, the ability to computationally consider hundreds of millions of peptides quickly (as implemented here) requires tens of gigabytes of memory and multi-gigahertz speed processors. Such resources are only now becoming widely accessible. The confluence of advancing sequencing and computational abilities has only recently made developing Display-Seq and the IMUNE algorithm feasible and neither would be worthwhile without the other. Given the validation demonstrated here in a small cohort of four IMUNE-identified motifs to diagnose CD using Display-Seq data and the expanding availability of sequencing and computational resources, we expect the Display-Seq method and the IMUNE algorithm to be of utility for identifying novel antibody biomarkers of disease and the corresponding antigens driving their production.

## Methods

This study and all experimental methods were approved by the University of California, Santa Barbara Human Subjects Committee, protocol CHNE-DA-PA-005-6N. All subjects gave written informed consent for the use of their specimens for research. All methods were carried out in accordance with UCSB’s Institutional Review Board approved guidelines.

### Identification of antibody binding peptides using bacterial display peptide libraries

#### Library screening of patient serum

31 serum samples from untreated CD, exhibiting a range of reactivities on tissue transglutaminase (TTG) and deaminated gliadin peptide (dGP) ELISA assays, and 28 HC (seronegative for TTG and dGP) serum samples from the Mayo Clinic (Rochester, MN) were used in this study. A large, high-quality, bacterial-display, random, 12-mer peptide library composed of 8 × 10^9^ independent transformants, was constructed using trinucleotide oligos to eliminate stop codons and normalize amino acid usage frequencies. The 12-mer peptide library is displayed on *E. coli* via the N-terminus of a previously reported, engineered protein scaffold (eCPX)^42^. To remove *E. coli* binding antibodies from CD serum samples prior to library screening, an induced culture of cells expressing the library scaffold alone was incubated with diluted sera. (*E. coli* strain MC1061 [FaraΔ 139 D(ara-leu)7696 GalE15 GalK16 Δ (lac)X74 rpsL (StrR) hsdR2 (rK−mK +) mcrA mcrB1] was used with surface display vector pB33eCPX.) eCPX cultures grown overnight at 37 °C with vigorous shaking (250 rpm) in LB (10 g tryptone, 5 g yeast extract, 10 g/L NaCl) supplemented with 34 μg/mL chloramphenicol (CM) and 0.2% glucose were collected by centrifugation, inoculated in fresh LB + CM, grown to an OD_600_ = 0.6, and induced for 1 hour at 37 °C with 0.02% wt/vol L(+)-arabinose. After induction, cells were centrifuged at 3,000 relative centrifugal force (rcf) for 5 min., washed once with cold PBST (PBS + 0.1% Tween 20), and resuspended in 1 mL PBST containing serum diluted 1:25 (1 × 10^10^ cells per depletion sample). Samples were incubated overnight at 4 °C with gentle mixing on an orbital shaker (20 rpm). Antibodies that bound to *E. coli* or the eCPX scaffold were removed by centrifugation of the incubated culture at 5,000 rcf for 5 min. twice, recovering the serum supernatant after each centrifugation. Depleted serum was stored at 4 °C for up to 2 weeks during use. The bacterial display peptide library was used to screen and isolate peptide binders to antibodies in individual serum samples through two rounds of Magnetic Activated Cell Sorting (MACS).

#### MACS #1

The first screen employed magnetic selection to enrich the library for antibody binding peptides as well as reduce the library size suitable for the subsequent screening steps. A frozen aliquot of the library containing 10^11^ cells (>10x the expected diversity) was thawed and inoculated into 500 ml LB + CM. After growth to an OD_600_ = 0.6 at 37 °C with 250 rpm shaking, the cells are induced with 0.02% wt/vol L(+)-arabinose for one hour using the same growth conditions. Cells (5 × 10^10^ per sample) were collected by centrifugation (3,000 rcf for 10 min.) and resuspended in 1 mL cold PBST. Prior to incubation with serum, cells were cleared of peptides that bind protein A/G by incubating cells with washed protein A/G magnetic beads (Pierce) at a ratio of one bead per 50 cells for 45 min. at 4 °C with gentle mixing. Magnetic separation for 5 min. (x2) was used to recover the unbound cells. Recovered cells from the supernatant are centrifuged, resuspended in diluted sera (1:25) and incubated for 45 min. at 4 °C with gentle mixing. Following serum incubation, cells were washed by centrifugation and resuspended in 1 mL cold PBST (x3). After the final resuspension, washed protein A/G magnetic beads were added at a ratio of one bead per 50 cells. After a 45 min. incubation with protein A/G beads at 4 °C with gentle mixing, a second magnetic separation isolated cells expressing peptides that bind to serum antibodies. The supernatant (unbound cells) was discarded and the separated cells/beads were washed with 1 mL cold PBST. 5 repeat washes were performed while the tube was being magnetized. After the last wash, the beads were resuspended in 1 mL of LB and inoculated into 25 mL LB + CM + glucose to suppress expression. The flask was grown overnight at 37 °C with shaking at 250 rpm. A 10 μL sample was removed prior to inoculation for dilution and plating on LB-agar to estimate the diversity of the enriched library.

#### MACS #2

A second round of magnetic affinity selection was carried out to further enrich the library for antibody binding peptides. After overnight growth of the first magnetic selection enriched libraries, cells were inoculated (>20x estimated diversity) at 1:50 into 10 mL LB + CM and grown to an OD_600_ = 0.6. After induction with arabinose for 1 hour, a volume of cells >20x the library diversity was centrifuged and resuspended in 100 μL cold PBST. Cells were again cleared of peptides that bind protein A/G by incubating cells with washed protein A/G magnetic beads (Pierce) at a ratio of one bead per 5 cells for 45 min. at 4 °C with gentle mixing. Magnetic separation for 5 min. (x2) was used to recover the unbound cells. Recovered cells from the supernatant are centrifuged, resuspended in diluted sera (1:25) and incubated for 45 min. at 4 °C with gentle mixing. Following serum incubation, cells were washed by centrifugation and resuspended in 100 μL cold PBST (x3). After the final resuspension, washed protein A/G magnetic beads were added at a ratio of one bead per 5 cells. After a 45 min. incubation with protein A/G beads at 4 °C with gentle mixing, a second magnetic separation isolated cells expressing peptides that bind to serum antibodies. The supernatant (unbound cells) was discarded and the separated cells/beads were washed with 100 μL cold PBST. 5 repeat washes were performed while the tube was being magnetized. After the last wash, the beads were resuspended in 1 mL of LB and inoculated into 5 mL LB + CM + glucose to suppress expression. The flask was grown overnight at 37 °C with shaking at 250 rpm. A 10 μL sample was removed prior to inoculation for dilution and plating on LB-agar to estimate the diversity of the final enriched library.

#### Analysis of enriched library using Fluorescence Activated Cell Sorting (FACS)

The following day, cells were analyzed for reactivity to the individual serum they were screened against to assess enrichment levels via FACS. After overnight growth of the MACS x 2 enriched library, cells are inoculated (>20x estimated diversity) at 1:50 into 5 mL LB + CM and grown to an OD_600_ = 0.6. After induction with arabinose for 1 hour, a volume of cells >20x the library diversity is centrifuged and resuspended in 50 μL diluted sera (1:25) for 45 min. at 4 °C. Cells are washed as described in the second round enrichment section (100 μL PBST) and resuspended in α-IgA-PE diluted 1:200 in 100 μL cold PBS. Following a 45 min. incubation at 4 °C, the cells were washed again and finally resuspended in 500 μL PBS for FACS analysis. Cells are analyzed for % of the cells with fluorescence signal greater than background (eCPX scaffold) by setting a gate to exclude ~99% of the signal from serum incubated with cells containing eCPX scaffold lacking peptide (negative control). Libraries with ~80% or greater enrichment (percent of cells that are above background/percent of peptides that bind serum antibodies) were processed for deep sequencing analysis.

### Amplicon Library Preparation for Next-Generation Sequencing

#### Preparation of amplicon library pools

Cells grown overnight after the second MACS enrichment were collected and plasmid was extracted using a plasmid miniprep kit (Qiagen). The random peptide region was amplified using a two-step PCR. For the first PCR step, the primers include adaptors specific to the Illumina sequencing platform with annealing regions that flank the random region (peptide library) of the eCPX scaffold. Forward Primer: TCGTCGGCAGCGTCAGATGTGTATAAGAGACAGxxxxx**CCAGTCTGGCCAGGG** Reverse Primer: **CCAGTACTACGGCATCAC**TGCTGTCTCTTATACACATCTCCGAGCCCACGAGAC Bolded regions anneal to the eCPX scaffold.

Products from the first PCR are purified after 25 rounds of PCR amplification (65 °C annealing temp) using Agencourt Ampure XP (Beckman Coulter) clean up beads. Resulting product is subjected to a second round of PCR using Illumina Nextera XT indexing primers (Illumina). These primers provide unique 8 base pair indicies on the 3 prime and 5 prime ends of the amplicons for tracking the sequences back to the sample used for screening and amplicon preparation. Amplicons are cleaned up as before after 12 rounds of PCR amplification (70 °C annealing temp). The final PCR product (amplicon) is analyzed using a DNA high sensitivity chip on a Bioanalyzer 2100 (Agilent) for purity, and DNA concentration was measured using DNA high sensitivity reagent on a Qbit instrument (Life Technologies). All samples were normalized to 4 nM and pooled together into a sequencing library.

#### Sequencing on Illumina NextSeq

After quality control for a single DNA amplicon of the correct size (High sensitivity chip using a Bioanalyzer 2100) and correct pool quantification is performed (High sensitivity Qbit analysis), the sample is diluted and loaded on to the NextSeq instrument. A 75 cycle high-output flow cell is used with single read (one direction) and dual indexing (both 5 prime and 3 prime indicies are sequenced). After sequencing is complete, the samples are automatically de-multiplexed using imputed sample identities with Illumina Nextera XT indicies.

### Generating a Non-Redundant Sequence List

Next-generation sequencing experiments can introduce many sources of error into the observed sequences, including expression bias, PCR bias and sequencing errors. Although it is likely that numerous observations of a peptide indicate that it is preferentially bound by a patient’s antibodies, deconvoluting the effects of antibodies’ affinity and concentration from the experimental error was outside the scope of this work. Therefore, all observations of identical peptides in a patient’s identified sequences are normalized to one. Given that the 12-mer, bacterial display peptide library contains 8 × 10^9^ members and was constructed using equimolar concentrations of trinucleotide codons, it was determined that sequences with three or fewer mutations were expected to be sequencing errors rather than unique sequences in the library. Supplemental Note 3 describes the algorithm to generate the non-redundant list.

### Identifying Statistically Significant Amino Acid Patterns

The IMUNE algorithm identifies putative autoimmune disease biomarkers by searching the peptide lists for patterns of amino acids that are statistically significant in many disease patients and not statistically significant in many control patients. An amino acid pattern is a series of three to five specific amino acids possibly interspersed with undefined positions, for a total length of three to ten residues. There are ~4.2 × 10^8^ possible patterns meeting these criteria. The upper bounds of five defined positions and ten total residues were selected based on an analysis of the expected diversity in a sample’s peptides and known antibody – antigen complexes as described in Supplemental Notes 1 and 2, respectively.

For each pattern, an expected number of observations in each sample’s sequences by random chance can be calculated. It is the product of the number of peptide sequences, the number of positions within a sequence where the pattern can fit and the percent usage of each of the pattern’s amino acids in the sample’s peptides (Supplemental Note 1, Eq. 1). Ideally, the percent usage of each amino acid in the unselected peptide library would be used to calculate the expected number of pattern observations, rather than the percent usage in a sample’s peptides. This would permit the calculation of the probability of seeing a pattern by random chance in *N* sequences from the library, rather than in the *N* selected peptides. Unfortunately, this data is not available. Therefore the observed usages were utilized.

IMUNE has three methods of identifying patterns that are significantly enriched in disease samples relative to control samples. They are:

(1) The Poisson Method: the expected observations can be compared to the actual observations of the pattern using Poisson distribution statistics (Supplemental Note 3, Eq. 3) to determine the probability that the pattern is present by random chance in the peptides. Patterns which are very statistically significant in many disease samples (e.g. *p* < 0.0001) and are not statistically significant in many control samples (e.g. *p* > 0.05) are retained for calculating motifs.

(2) The Standard Deviation Method: the average enrichment and sample standard deviation are calculated for the control samples. Patterns are retained if they are a sufficient number of standard deviations (e.g. 5) above the average of the controls in a sufficient fraction of the disease samples.

(3) Receiver operating characteristic (ROC) curve: Patterns are retained if their enrichments rank order such that there is a point where they meet specified sensitivity and specificity (e.g. 50% and 100%) thresholds.

IMUNE was run five different ways in this study (all ran simultaneously and finished in the 9 hr. time stated in the Results): (*i*) using the Poisson method to find patterns with *p* < 0.0001 in 50% of CD samples and *p* > 0.0313 in 100% of HC samples, (*ii*) using the Standard Deviation method to find patterns at least four standard deviations above the average of the HC samples in at least 50% of the CD samples, (*iii*) using the Poisson method to find patterns with *p* < 0.0001 in 50% of HC samples and *p* > 0.0313 in 100% of the CD samples, (*iv*) using the Standard Deviation method to find patterns at least four standard deviations above the average of the CD samples in at least 50% of the HC samples and (*v*) with *p* < 0.0001 in 80% of the combined CD and HC samples. A specificity *p* value of 0.0313 was used because it corresponds to a 50% chance of one of 16 CD samples having a pattern that enriched by random chance (i.e. 0.50/16 = 0.0313). The identified motifs from runs *i* and *ii* and runs *iii* and *iv* were highly similar and treated as a single set of motifs for the Results.

### Creating Motifs via Pattern Clustering

Once all patterns have been evaluated for statistical significance, those that were retained are clustered together to generate the motifs using a hierarchical and iterative process. First, “motif seeds” are identified, where a motif seed is any pattern that does not contain another statistically significant pattern. A pattern that does contain another statistically significant pattern is considered a “child pattern” of that motif seed. Motif seeds may have zero or more child patterns and each child pattern has one or more motif seeds to which it belongs (e.g. PEQPxP is a child pattern of motif seeds PEQP and PEQxxP).

Next, the PAM30^43^ similarity matrix is used to align motif seeds to one another. For every pair of motif seeds, all possible alignment frames are considered and the highest scoring alignment is selected. A score is calculated for a given alignment by comparing the defined amino acids in each motif seed to the corresponding residues in the other pattern. If neither motif seed has a specified amino acid at a given position, no score is included for that spot. Two motif seeds are considered related if they have an alignment score of at least five. Once all motif seeds have been compared, they are ordered by the total number of patterns to which they are connected (i.e. the seed’s child patterns, its related motif seeds and their child patterns). The motif seeds are then used to generate preliminary motifs.

Constructing a motif for a motif seed begins with an alignment of all patterns connected to the motif seed. Each pattern also has a weight associated with it related to either its enrichments or observations in the samples. In all cases, we used the summed enrichments among all samples a pattern should be sensitive to (i.e. the CD samples). The frequencies of each amino acid in each column of the alignment are calculated and compared to their expected frequencies. Amino acids are included in the regular expression for a motif if they occur at least 1.5 times more often than expected and are very statistically significant (i.e. *p* < 0.0001).

Initial results demonstrated that the preliminary motifs contained much unidentified redundancy. Similar motifs are identified by aligning them to one another using the PAM30 similarity matrix, as was done for the motif seeds. The score for a pair of positions in a motif alignment is calculated as the highest score from comparing the most enriched amino acid in each motif to every amino acid at that position in the other motif. Two motifs are considered related if they have alignment scores of at least ten. Once all motifs have been compared to one another, they are re-ordered based on the total number of pattern contributions as was done for the motif seeds. Numerous values were tested for the “relatedness” thresholds of five and ten for motif seeds and motifs, respectively. These values were selected on the basis of best identifying redundant motifs without combining visually dissimilar motifs based on manual inspection.

Finally, several motif lists are generated. This includes a redundant list containing the calculated motif for every motif seed. Additionally, non-redundant lists are output for each of the statistical methods regardless of which was used to identify the patterns. For each statistical method, the motifs are rank ordered based on their specificities and sensitivities according to the method. Each motif is then sequentially included in the list so long as its motif seed was not used in making another motif that was previously included in the list. Even after these steps, the method specific lists contain motifs that are highly similar to one another. The values presented in Supplemental Table 2 are the total numbers of motifs in each list, but manual inspection reduced them to the nine CD and five HC motifs in the Results.

## Additional Information

**How to cite this article**: Pantazes, R. J. *et al*. Identification of disease-specific motifs in the antibody specificity repertoire via next-generation sequencing. *Sci. Rep.* **6**, 30312; doi: 10.1038/srep30312 (2016).

## Supplementary Material

Supplementary Information
