# Supplementary material for: Identification of disease-specific motifs in the antibody specificity repertoire via next-generation sequencing
Source: Sci Rep. 2016 Aug 2;6:30312. doi: 10.1038/srep30312 (PMC4969583; doi:10.1038/srep30312)
Supplement: Supplementary Information [file srep30312-s1.pdf]

Identification of disease-specific motifs in the antibody specificity repertoire via next-generation sequencing: Supplemental Information

Authors: Robert J. Pantazes, Jack Reifert, Joel Bozekowski, Kelly N. Ibsen, Joseph A. Murray, Patrick S. Daugherty

Supplemental Figure 1. MEME-identified multiple motifs similar to QPEQPF[PS]E.
a) WebLOGO plots, using bits to determine character heights, of four MEME-identified motifs which correspond to the dominant IMUNE motif of QPEQPF[PS]E. *b)* enrichments of these motifs in CD samples 77, 88, and 89. These samples are not highly enriched in at least one motif and moderately enriched in another as they were for the IMUNE-identified motifs.

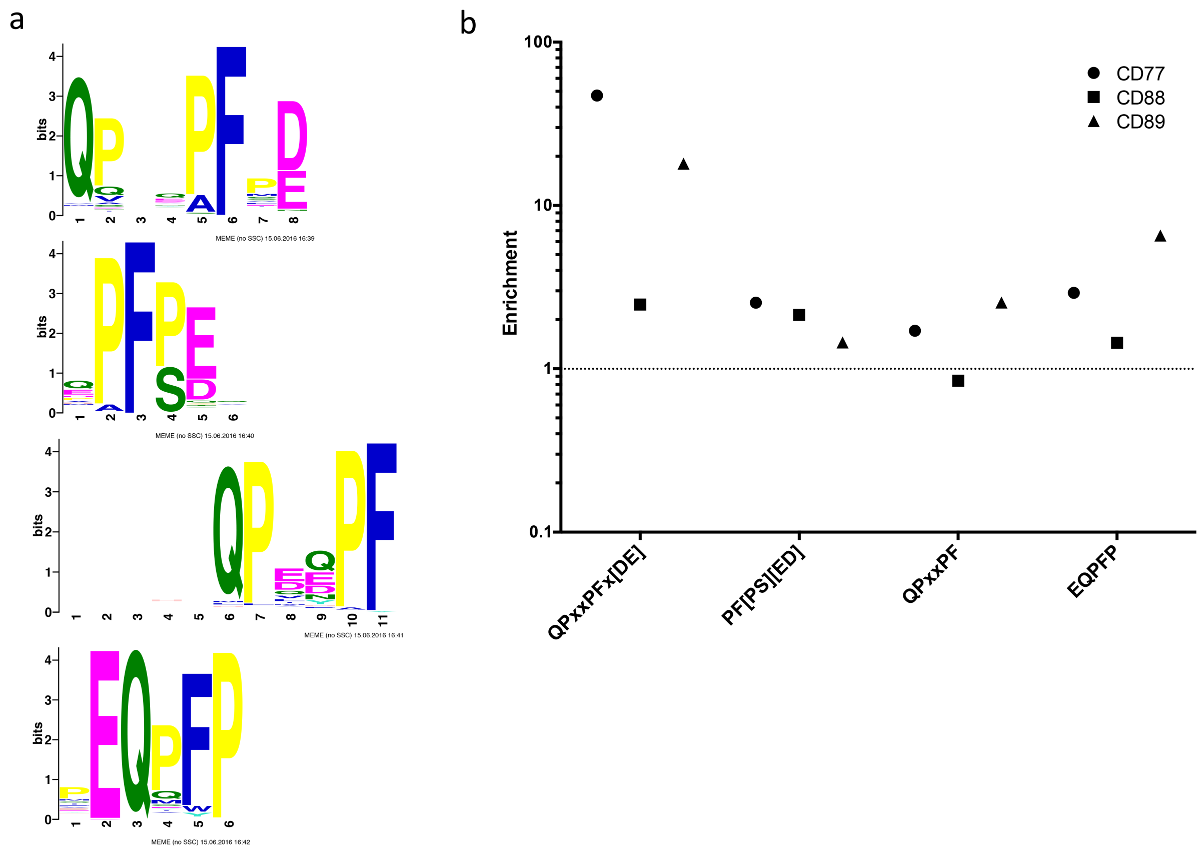

Supplemental Figure 2. Similar MEME- and IMUNE-identified motifs. MEME identified six non-gliadin-like motifs that were also identified by IMUNE. However, MEME failed to identify motifs similar to WSP[YF][VTIL] and D[FY][PLTIV]xxYD; motifs found by IMUNE that were considered as possible biomarkers (Fig. 2).

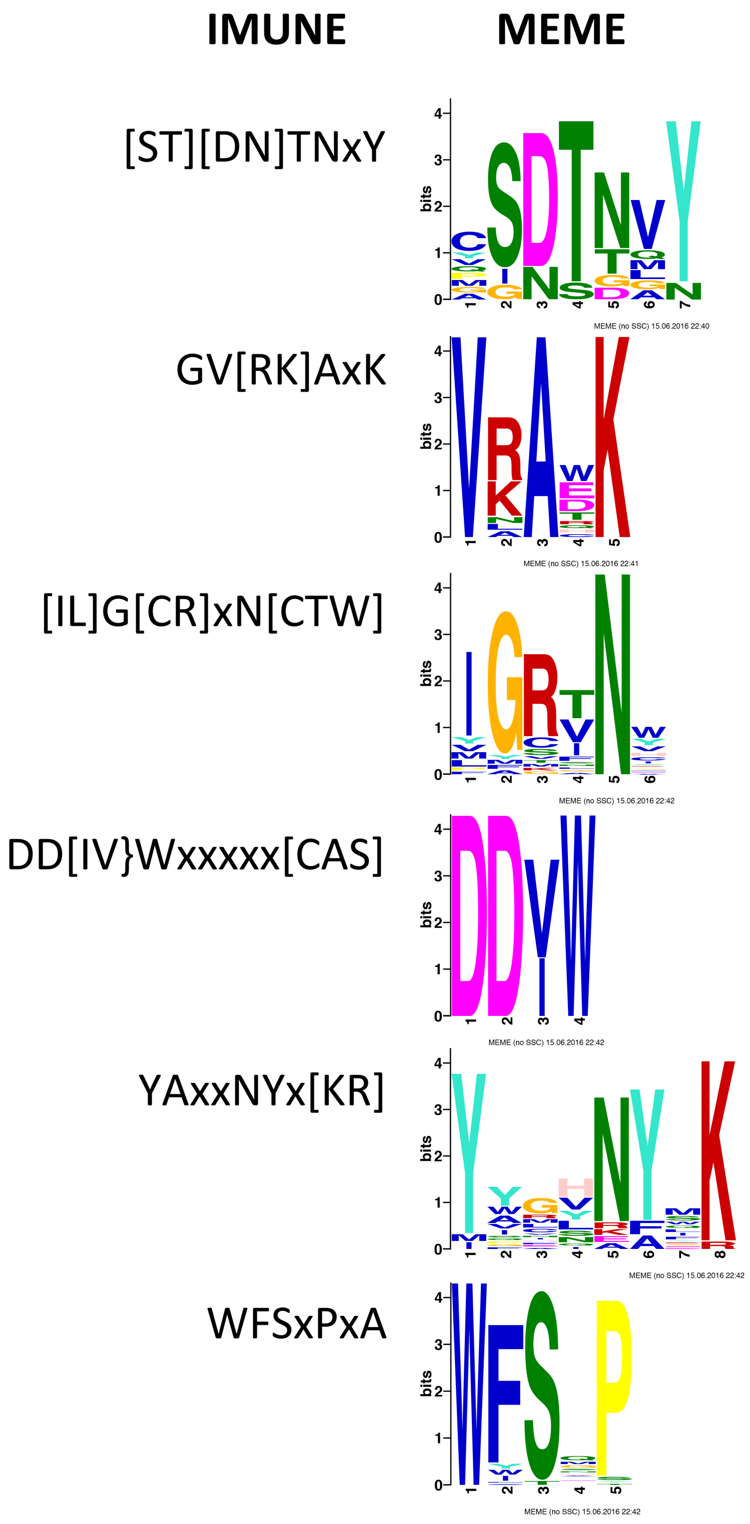

Supplemental Figure 3. MEME identified multiple non-specific motifs. MEME found 6 motifs (*a*) that are poor candidate biomarkers because they are not highly enriched in many CD samples and / or are highly enriched in several HC samples (*b*).

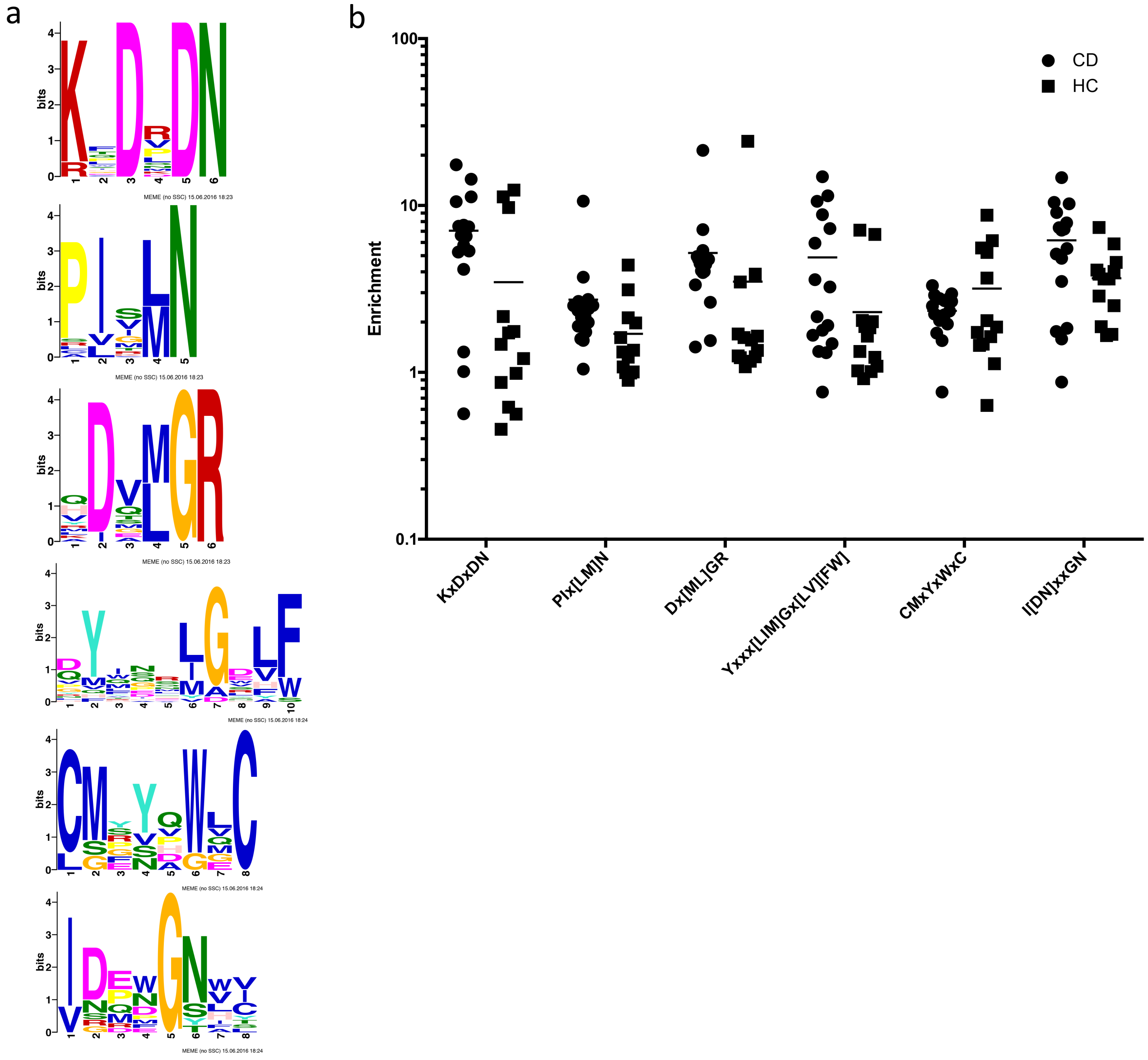

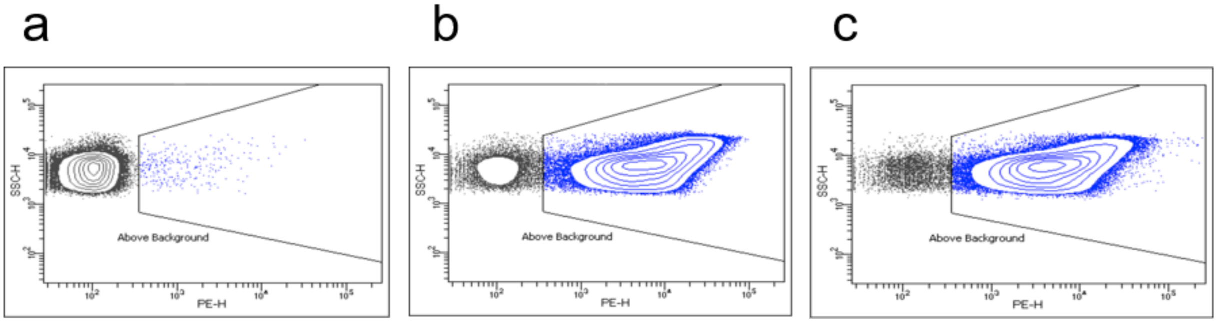

Supplemental Figure 4. Representative FACS enrichment plots. FACS was used to evaluate binding to the library after two rounds of MACS. *a)* representative plot for the binding of depleted serum antibodies to the eCPX scaffold alone (i.e. background fluorescence). *b)* and *c)* examples of moderately (~78% above background, validation sample CD69) and highly (~95%, validation sample HC4) enriched libraries.

Supplemental Table 1. Unique and total sequences identified for CD and HC samples used for motif discovery.

Sample	Total Sequences	Unique Sequences
**CD74**	6.51E+06	2.42E+06
**CD75**	1.02E+07	2.83E+06
**CD77**	1.44E+07	4.66E+06
**CD78**	5.45E+06	2.39E+06
**CD80**	1.02E+07	2.64E+06
**CD81**	9.37E+06	3.48E+06
**CD83**	1.14E+07	4.51E+06
**CD84**	7.90E+06	2.89E+06
**CD86**	1.22E+07	3.59E+06
**CD87**	7.54E+06	1.51E+06
**CD88**	7.31E+06	2.32E+06
**CD89**	9.44E+06	2.80E+06
**CD90**	5.86E+06	2.12E+06
**CD91**	1.20E+07	3.37E+06
**CD93**	9.85E+06	3.16E+06
**CD94**	1.07E+07	2.66E+06
**HC13**	7.30E+06	2.26E+06
**HC14**	9.82E+06	2.40E+06
**HC15**	3.48E+06	1.68E+06
**HC16**	7.47E+06	2.28E+06
**HC17**	5.52E+06	1.87E+06
**HC18**	1.00E+07	3.15E+06
**HC19**	9.90E+06	4.39E+06
**HC20**	6.61E+06	2.64E+06
**HC21**	1.07E+07	3.63E+06
**HC22**	1.08E+07	3.21E+06
**HC23**	4.46E+06	1.76E+06
**HC24**	7.92E+06	2.24E+06
**HC25**	9.29E+06	2.92E+06

Supplemental Table 2. IMUNE Algorithm Results for Celiac Disease. As described in the Online Methods, IMUNE can use multiple statistical tests to both identify patterns that are significantly different between two populations as well as to prioritize motifs when generating non-redundant motif lists. Here, the Poisson and Standard Deviation methods were each used to identify patterns that were specific (100%) and sensitive (≥ 50%) to both the CD and HC populations. Only the Poisson method was used to identify patterns sensitive to both populations (≥ 80%) because the Standard Deviation method *must* compare between two populations. However the patterns are identified in a given assessment, IMUNE generates different non-redundant motif lists prioritized for each statistical method.

Assessment	Specific and Sensitive to	Pattern Identification Method	Patterns	Motif Seeds	Unique Poisson Motifs	Unique Standard Deviation Motifs
1	CD	Poisson	11153	8571	454	421
2	CD	Standard Deviation	16279	5723	267	253
3	HC	Poisson	646	592	26	27
4	HC	Standard Deviation	3024	1826	77	70
5	Common	Poisson	68879	9377	1321	N/A

Supplemental Note 1. Limiting Patterns and Motifs to Five Defined Positions

The peptide library used in this work was composed of random 12-mer peptides constructed using synthetic tri-nucleotide codons such that every amino acid has an equal probability of occurring in the peptides (Online Methods). Therefore, the expected observations E of a pattern of n defined positions in L total residues occurring in N peptides is:

$$E = (N)\left(\frac{1}{20}\right)^{n}(12 - L + 1) \quad (1)$$

where the third term is the number of positions within a peptide where the pattern may occur. Given a maximum pattern length of 10 total residues (Supplemental Note 2) and $N = 1.51\times10^{6}$, E equals 1.41 for $n = 5$ and 0.07 for $n = 6$. Therefore, IMUNE was limited to using no more than 5 defined positions in patterns and motifs for calculating enrichments to ensure that the expected observations number exceeded one for all patterns and motifs in all samples. Although this is not strictly necessary, the authors felt that an enrichment of 20 was more meaningful when it came from 20 observations with one expected by random chance than two observations with 0.1 expected observations.

Supplemental Note 2: Checking for Linear Epitopes in Antibody-Antigen Complexes.

We analyzed 125 non-redundant antibody-antigen complexes from the Protein Data Bank[1]. All structures had resolutions no worse than 2.5 Å and bound a protein antigen. We defined the epitopes of the antigens to be every residue with at least one heavy atom (i.e. non-hydrogen) within 4.5 Å of a heavy atom in the antibody's complementarity determining regions (CDRs). The threshold of 4.5 Å and the identification of the CDRs are consistent with previous projects[2,3]. We found that 122 / 125 (97.6%) of the antigens had at least five residues contacting the antibody within a window of ten sequential residues. Thus, almost all epitopes contain a core linear component. Expanding beyond ten residues did not improve the results, and therefore we defined the maximum length of a pattern in the IMUNE algorithm as ten residues.

Supplemental Table 3. Unique and total sequences for the validation CD and HC samples.

Sample	Total Sequences	Unique Sequences
**CD56**	7.60E+06	2.60E+06
**CD57**	7.41E+06	2.42E+06
**CD58**	8.00E+06	2.37E+06
**CD61**	9.42E+06	2.86E+06
**CD62**	8.69E+06	2.87E+06
**CD63**	9.47E+06	2.05E+06
**CD64**	7.74E+06	2.76E+06
**CD65**	8.15E+06	3.02E+06
**CD66**	8.31E+06	3.25E+06
**CD67**	8.18E+06	2.75E+06
**CD69**	8.89E+06	3.09E+06
**CD70**	5.95E+06	2.66E+06
**CD71**	7.12E+06	1.90E+06
**CD72**	1.03E+07	3.60E+06
**CD95**	9.67E+06	2.64E+06
**N2**	7.39E+06	2.18E+06
**N3**	6.80E+06	2.06E+06
**N4**	7.92E+06	2.45E+06
**N5**	5.96E+06	2.18E+06
**N7**	6.26E+06	2.23E+06
**N10**	8.43E+06	3.12E+06
**N12**	7.56E+06	2.75E+06
**N26**	9.83E+06	3.23E+06
**N27**	1.04E+07	3.14E+06
**N28**	9.88E+06	2.78E+06
**N29**	9.40E+06	3.20E+06
**N30**	7.81E+06	2.40E+06
**N31**	8.00E+06	2.86E+06
**N32**	7.93E+06	2.95E+06
**N33**	1.23E+07	3.58E+06

Supplemental Note 3. Generating a non-redundant sequence list.

The NGS experiments generated FASTQ formatted files of DNA sequences. All results for this project were demultiplexed, but the algorithm is able to analyze multiplexed data. The only difference in the implementation is that before a sequence from the multiplexed data can be analyzed, a sample-specific barcode must be precisely identified. Once the barcode is found, the sequence is assigned to the corresponding sample, the barcode information is stripped off and sequence identification proceeds as described below.

The DNA sequences have known constant annealing regions bordering the peptides. The algorithm first identifies the start and end of the upstream constant region, allowing up to 25% insertions, deletions and/or mutations (e.g. if the annealing region is 16 nucleotides in length, no more than four total insertions, deletions and mutations are permitted). Next, the start and end of the downstream annealing region are found, again allowing up to 25% errors. The search for the downstream annealing region starts at the expected position from the end of the upstream region (i.e. 36 nucleotides), and progressively searches both longer and shorter lengths until the region is found. If either annealing region is not located, the algorithm fails to identify a peptide from the DNA sequence. Once both annealing regions are located, the peptide sequence can be retrieved by translating the intervening DNA, with sequences containing stop codons permitted. Sequences that do not correspond to a peptide of proper length (i.e. DNA sequences that are not 36 base pairs) are not retained. Identified peptide sequences are stored in a unique file for each sample.

Once all peptide sequences are identified from the next generation sequencing FASTQ files, the non-redundant peptide lists are created. First, for each sample all exactly identical sequences

are combined. This is done using an efficient recursive algorithm that searches lists of peptides with identical amino acids at checked positions. Search branches are retained only so long as they contain multiple peptides. Branches that have checked all positions while still containing multiple peptides have found identical sequences, which are combined into a single entry while retaining the total number of observations. The time requirements for this step are linear for both the length and number of peptides.

The peptide library construction dictates the maximum number of mutations two peptides may have to be considered identical. The 12-mer, bacteria-displayed peptide library used in this manuscript contains ~7.6×10^9 members and was constructed using synthetic trinucleotide codons with an equal probability of each amino acid occurring. Poisson distribution statistics indicate that the probability of having exactly *N* observations of an event when *E* are expected is:

$$Prob(E,N) = \frac{E^N e^{-E}}{N!} \quad (2)$$

And the probability of at least *N* observations when *E* are expected is:

$$p(E,N) = 1 - \sum_{i=0}^{N-1} Prob(i,E) \quad (3)$$

If two peptides are identified as having *n* identical positions, than we can calculate the expected number of library peptides that contain those specific residues at those positions. Because there was an equal probability of incorporating each amino acid at each position during library construction, every peptide is equally likely to occur in the library. The expected observations of a peptide with *n* specific positions is

$$E = (7.6\times10^9)\left(\frac{1}{20}\right)^n \quad (4)$$

E is 1.86×10^{-6} for $n = 12$, 3.71×10^{-5} for $n = 11$, 7.42×10^{-4} for $n = 10$ and 1.48×10^{-2} for $n = 9$. The corresponding p values calculated with equation 3 for observing two or more such peptides in the library are $< 1\times10^{-10}$, 6.88×10^{-10}, 2.75×10^{-7} and 1.08×10^{-4} respectively. When determining the statistical significance of amino acids patterns in the peptides bound by a sample's antibodies, a strict p value of 1×10^{-4} is used. To be consistent with that value, peptides with 9 or more identical positions (i.e. three or fewer mutations) are combined into a single consensus sequence.

Efficiently identifying peptides with several mutations is more complex than finding identical sequences, as the number of comparisons required is $O(N^2)$. Fewer comparisons can be achieved by only comparing sequences that are known to be similar to one another, such as having n exactly identical positions. In such an instance, the number of comparisons is reduced to $O\left((20^n)\left(\frac{N}{20^n}\right)^2\right) = O\left(\frac{N^2}{20^n}\right)$. However, it is possible for sequences to contain mutations within the n positions that were required to exactly match. Therefore, sufficient unique groups of positions must be used to guarantee that all peptides with three or fewer mutations are compared to one another. The IMUNE algorithm optimally determines n to find all mutated sequences using a minimum number of comparisons.

Once all mutant peptides are identified, the peptide list is reorganized by the total number of observations (i.e. identical and mutant). The list is then traversed in this order, generating consensus peptides using the most abundant amino acids at each position and allowing stop codons. The consensus peptides retain the total number of observations of each contributing peptide. Once a peptide has contributed to a consensus peptide, that peptide is removed from the peptide list and not used in any other consensus peptide. Therefore, the consensus peptides

generated are dependent on the ordering of the peptide list. Consider the example of peptide *A*, which has two mutations with peptide *B* and two different mutations with peptide *C*. If the peptides were ordered *A, B, C*, than peptides *B* and *C* would both contribute to the consensus peptide made from peptide *A*. If they were ordered *B, A, C*, than peptide *A* would contribute to peptide *B*'s consensus peptide but peptide *C* would not as it has four mutations with *B*.

The identification of mutated peptides and the generation of consensus sequences is iteratively repeated until no additional mutants are identified (i.e. every peptide has more than three mutations from every other peptide). Once this has been achieved, peptides that contain stop codons are removed from the list. The construction of the peptide library guarantees that such peptides could not have been selected by a patient's antibodies. Therefore, peptides with stop codons represent known experimental error, most likely sequencing errors, that can be safely removed prior to computational analysis. A unique peptide list is generated for each sample.

References

1. Berman, H. M. *et al.* The Protein Data Bank. *Nucleic Acids Res.* **28,** 235–242 (2000).

2. Pantazes, R. J. & Maranas, C. D. MAPs: a database of modular antibody parts for predicting tertiary structures and designing affinity matured antibodies. *BMC Bioinformatics* **14,** 168 (2013).

3. Pantazes, R. J., Saraf, M. C. & Maranas, C. D. Optimal protein library design using recombination or point mutations based on sequence-based scoring functions. *Protein Eng. Des. Sel.* **20,** 361–373 (2007).
